# Association of Hallucinogen Use With Psychosocial Outcomes: Secondary Analysis of the Spinal Cord Injury Model Systems Database

**DOI:** 10.46292/sci24-00082

**Published:** 2025-11-18

**Authors:** Kayleigh Crane, Angela Hanks Philippus, Jennifer Coker, Jesse L. Kowalski, Kimberley R. Monden

**Affiliations:** 1 College of Medicine, Central Michigan University, Mount Pleasant, Michigan; 2 Department of Rehabilitation Medicine, University of Minnesota Medical School, Minneapolis, Minnesota; 3 Research Department, Craig Hospital, Englewood, Colorado

**Keywords:** depression, hallucinogens, quality of life, spinal cord injuries, substance use

## Abstract

**Background:**

Individuals with spinal cord injuries (SCI) experience depression at rates up to 5 times higher than the general population. Although research on hallucinogens for treating depression is growing, no studies have specifically focused on individuals with SCI.

**Objectives:**

To identify factors associated with hallucinogen use and explore the relationship between hallucinogen use and psychosocial outcomes (i.e., depression and quality of life [QOL]) among individuals with SCI.

**Methods:**

This exploratory, retrospective cross-sectional study design used data from 9976 participants in the Spinal Cord Injury Model Systems (SCIMS) National Database (NDB) between 2016 and 2021. The study outcomes were depression (Patient Health Questionnaire–9 [PHQ-9]) and QOL (Satisfaction with Life Scale [SWLS]). Hallucinogen use, measured by the WHO ASSIST 2, was dichotomized into some use versus no use.

**Results:**

Overall, 65 participants (0.7%) reported some hallucinogen use in the prior 3 months. Multivariable logistic regression analysis showed that younger age, male sex, fall(s) in the previous year, and sleep disturbances were all associated with hallucinogen use. In multivariable linear regression, hallucinogen use was significantly associated with higher PHQ-9 scores (effect size [ES] 0.558) but was not significantly related to SWLS scores (ES 0.149).

**Conclusion:**

This is the first study to identify demographic, injury, and psychosocial factors associated with hallucinogen use among individuals with SCI and to explore its relation to depression and QOL. These findings provide a foundation for future, fully powered studies aimed at determining the effect of hallucinogen use in the SCI population.

Individuals with traumatic spinal cord injury (SCI) have depression rates as high as 1 in 5, nearly 4 times the national average.^1^ Prolonged depressive symptoms indicative of treatment resistance are also reported in individuals with SCI, with 23% reporting persistent mood disturbance over time.^2^ Recent studies suggest that hallucinogens may offer an alternative therapy for major depressive disorder.^3^ Clinical evidence suggests that psychedelics such as psilocybin may aid in treating depression, especially in treatment-resistant cases.^4^–^8^

Therapeutic benefits of hallucinogens have been reported for a variety of psychiatric conditions including mood, substance use, personality, sleep, and eating disorders. Hallucinogens, specifically lysergic acid diethylamide (LSD) and psilocybin, have also been investigated regarding their role in management of chronic pain.^6^ In individuals with SCI, chronic neuropathic pain is present in 40% to 50% of individuals, leading to interferences with daily activities and influence quality of life (QOL).^9^,^10^ Despite reported therapeutic benefits, most remain Schedule I drugs (except ketamine), classified as having a high potential for abuse and dependence.^11^ As of June 2023, the United States Food and Drug Administration (FDA) approved clinical trials investigating psychedelics like psilocybin, LSD, and methylenedioxymethamphetamine (MDMA) to treat mood, anxiety, and substance use disorders.^6^,^12^–^14^

The FDA categorizes hallucinogens based on how they affect the brain: (1) psychedelics (e.g., LSD, psilocybin) target serotonin 2A receptors (5-HT2A)^15^; (2) dissociatives (e.g., ketamine) act as N-methyl-Daspartate (NMDA) receptor antagonists^16^; and (3) substances like MDMA, ibogaine, and salvia have unique effects.^17^ Research indicates that psilocybin, derived from “magic mushrooms,” may alleviate depression symptoms by promoting brain plasticity, although its exact mechanisms remain unclear.^8^,^18^ Activation of the 5HT2A receptor is thought to play a role in these effects, through modulation of altered 5-HT signaling found to contribute to depressive disorders.^19^,^20^ Following SCI, 5-HT supply is lost in the descending tracts, and varying degrees of neuroplasticity occur with these receptors.^21^ The 5-HT receptors in the spinal cord have a dual action in pain with both a pro- and antinociceptive inflammatory state that can occur when stimulated. A study reviewing online forums of individuals with SCI using hallucinogens identifies a serotonin-like syndrome response amongst users given the alteration in 5-HT receptors in the spinal canal. Impairments in 5-HT signaling in individuals with SCI, mechanisms that also contribute to depression and pain, suggest hallucinogen-induced modulation of 5-HT signaling could support mood and QOL in this population.^22^,^23^

However, research on psychedelic use among individuals with physical disabilities is limited.^24^ In 2022, *Frontiers in Psychiatry* urged for the inclusion of individuals with physical and sensory disabilities within the emerging research of psychedelic therapies, as they highlighted the disinclusion of this group and the importance the research being conducted on outcomes in these populations.^24^ Currently, only one study is listed on ClinicalTrials. gov regarding SCI and hallucinogens, highlighting the need for emphasized research in this domain. This exploratory study aimed to identify factors associated with hallucinogen use and assess its relationship with depression and QOL among individuals with SCI. We hypothesized that hallucinogen users would report fewer depressive symptoms and improved QOL compared to nonusers.

## Methods

### Study design

An exploratory retrospective cross-sectional design was used to analyze data from individuals with SCI in the Spinal Cord Injury Model System (SCIMS) National Database (NDB), funded by the National Institute on Disability, Independent Living, and Rehabilitation Research (NIDILRR). Study activities were reviewed by the University of Minnesota Institutional Review Board (IRB) (STUDY00019212), and the study was determined “not human research,” meaning that the study activities would not be subject to the requirements of DHHS and FDA regulated research involving human subjects. Eighteen SCIMS centers contribute data on newly acquired SCI cases for over 36,990 individuals, covering acute, rehabilitation, and follow-up stages, with follow-ups extending up to 50 years. Injury data are sourced from medical records, with follow-ups at 1 year, 5 years, and every 5 years thereafter. Our analysis specifically targeted cases from the 2016-2021 grant cycle, as the hallucinogen use variable was introduced then.

### Participants

Participants are eligible for the SCIMS NDB if they acquire a traumatic SCI, are admitted to a SCIMS rehabilitation hospital within a year, live in the catchment area, complete inpatient rehabilitation, have not regained normal neurologic status, and provide informed consent.

We included participants aged 18 or older who completed a recent follow-up interview between October 31, 2016, and September 1, 2021. For those with multiple interviews, we selected the most recent interview to ensure independent observations. Only participants with data on hallucinogen use were included.

**Figure 1.**
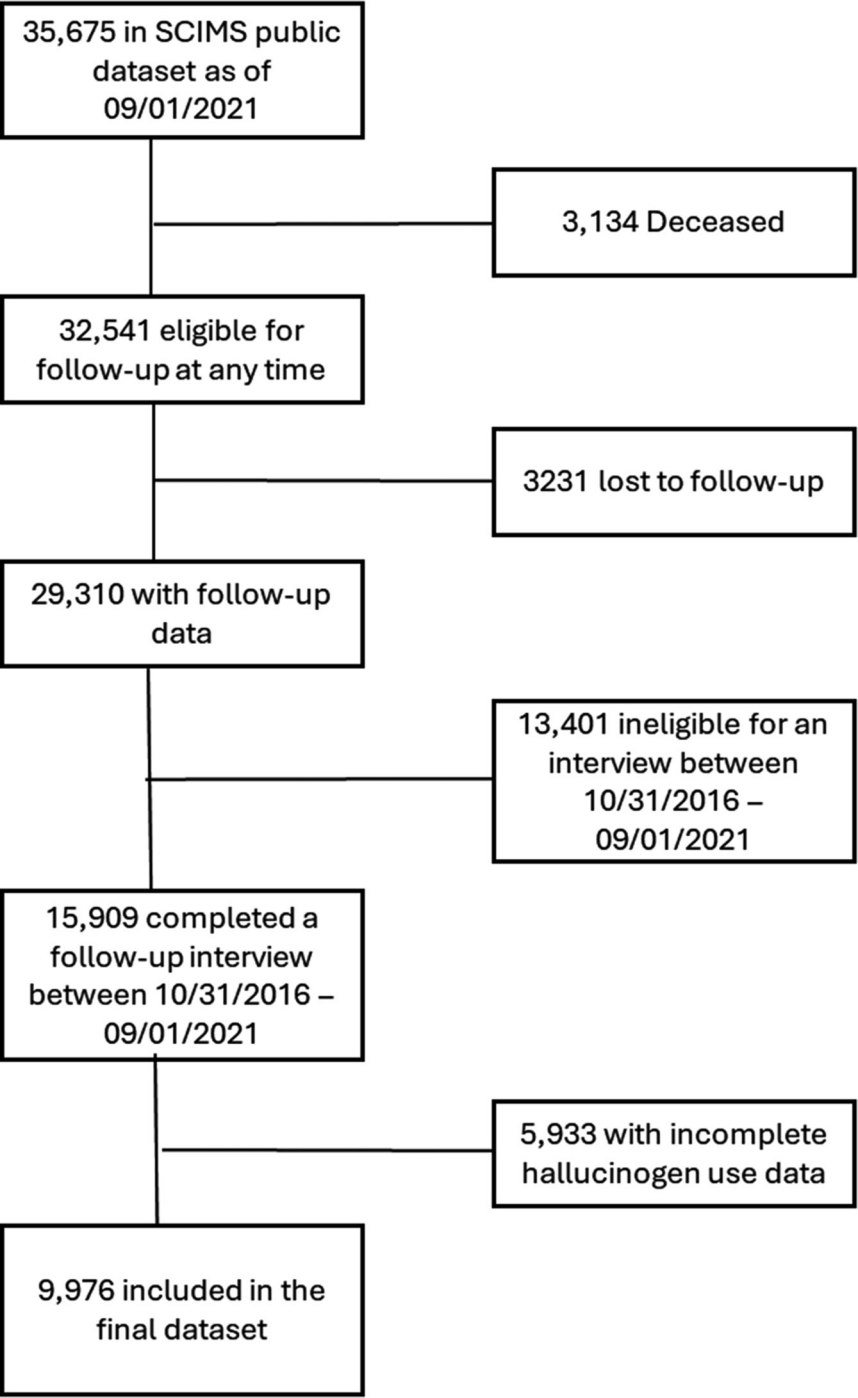
Enrollment flowchart.

As of September 1, 2021, the SCIMS public dataset contained 35,675 individuals, with 3134 coded as deceased; this left 32,541 eligible for follow-up. Among these, 29,310 had valid follow-up data, and 15,909 completed interviews between October 31, 2016, and September 1, 2021. After excluding those with incomplete hallucinogen use data (*n* = 5933), the final sample included 9976 individuals (see **Figure 1**).

### Variable definition

The outcomes of interest were depression symptoms and QOL. Depression symptoms were assessed using the Patient Health Questionnaire–9 (PHQ-9),^25^ where participants rated the frequency of 9 symptoms over the past 2 weeks on a scale from 0 (*not at all*) to 3 (*nearly every day*). Scores range from 0 to 27, with higher scores indicating more severe depression symptoms. QOL was assessed with the Satisfaction with Life Scale (SWLS),^26^ where participants rated their agreement with 5 statements from 1 (*strongly disagree*) to 7 (*strongly agree*). Scores range from 5 to 35, with higher scores indicating higher QOL.

The primary predictor, hallucinogen use, was measured using the World Health Organization Alcohol Smoking and Substance Involvement Screening Test-2 (WHO ASSIST 2),^27^ which asks participants to rate their frequency of use of 9 substances in the past 3 months from 0 (*neve*r) to 4 (*daily or almost daily*). For the hallucinogen item in particular, several examples of hallucinogens were provided, including LSD, acid, mushrooms, PCP, Special K, and Lucy. For this study, hallucinogen use was dichotomized as “some use” (WHO ASSIST 2 psychedelic item > 0) and “no use” (WHO ASSIST 2 psychedelic item = 0).

The sample was described using demographic and injury characteristics, including age, time post injury, education level (less than high school [HS], HS diploma or GED, greater than HS), income (<$50,000, ≥$50,000), race (white, black, other/multiracial), sex (male, female), marital status (married, single/never married, previously married), and occupational status (employed/ student, not employed). Neurologic impairment was classified using the American Spinal Injury Association Impairment Scale (AIS) as either complete (AIS A) or incomplete (AIS B, C, D).

We evaluated psychosocial factors related to hallucinogen use, including pain interference in the past 4 weeks (none, some, a lot), rehospitalization in the prior year (none, some), sleep disturbance in the prior year (none, some), falls in the previous year (none, some), and perceived health status (good, poor).^28^

### Statistical analysis

All statistical analyses were performed in JMP Pro version 17 at a significance level of *α* = 0.05 unless specified otherwise. Demographic and injury characteristics were summarized for the full sample and by hallucinogen use group, reporting means and standard deviations for continuous variables and frequencies and percentages for categorical variables.

Bivariate logistic regressions identified factors associated with hallucinogen use. Pearson chi-square tests were used for categorical covariates and analysis of variance (ANOVA) for continuous covariates. Non-normally distributed variables were tested with the Kruskal-Wallis test. Factors with *P* < .20 were included in the multivariable logistic regression, with both unadjusted and adjusted odds ratios (OR) and 95% confidence intervals (CI) reported.

Linear regression analyses were conducted to identify how hallucinogen use was associated with depression symptoms (PHQ-9) and QOL (SWLS). Bivariate analyses examined the relationship of each demographic, injury-related, and psychosocial factor (including hallucinogen use) on each outcome using ANOVA for categorical covariates and linear regression for continuous covariates. The mean, standard error of the mean (SEM), and model *P* value are reported for categorical factors, and regression coefficient, SEM, and model *P* value are reported for the continuous factors. Hallucinogen use plus any significant variables identified in bivariate analyses were subjected to subsequent multivariable analysis. The total amount of variance explained by each model was quantified using the adjusted *R*^2^ statistic for both the unadjusted and adjusted models. The adjusted relationship between hallucinogen use and each outcome was quantified as the estimated mean difference between users and nonusers and evaluated with a *t* statistic; uncertainty in estimates was quantified with a 95% CI or 95% confidence level (CL). Effect sizes (ES) were computed as the mean difference divided by the root mean square error from the model. ES of 0.2, 0.5, and 0.8 are typically considered small, medium, and large.^29^ For each adjusted model, the ranked significance of each covariate was characterized by the *F* statistic and its corresponding *P* value.

## Results

### Sample characteristics

The final dataset contained information from 9976 adults with SCI; sample characteristics are described in **Table 1**. The sample comprised primarily males, people who identified as white and not Hispanic, were married or previously married, and those with a household income of less than $50,000 a year. The median time since injury was 11 years, and the average age was 51 years. Nearly 90% of the sample had a high school education or greater, and most were unemployed. Almost 60% of the sample had incomplete injuries, and 99% reported no hallucinogen use over the previous 3 months. Sixteen percent of the sample met the clinical definition of moderate to severe depression (PHQ-9 scores >9),^25^ and 66% reported feeling neutral, satisfied, or extremely satisfied with life (SWLS score >19).^26^

**Table 1.** Sample sociodemographic and injury-related characteristics (*N* = 9976)

Categorical variables	*n*	%
Sex		
Male	7871	78.9
Female	2101	21.1
(Missing)	(4)	
Education		
Less than high school	895	9.2
High school diploma or GED	4717	48.6
Greater than high school	4091	42.2
(Missing)	(273)	
Income		
Less than $50k	5078	61.2
$50k or more	3222	38.8
(Missing)	(1676)	
Marital status		
Married	4146	41.7
Not married	3640	34.8
Previously married	2338	23.5
(Missing)	(32)	
Employment status
Employed/Student	2585	27.2
Not employed	6916	72.8
(Missing)	(475)	
Race		
White	6968	72.1
Black	2145	22.2
Other, multiracial	557	5.8
(Missing)	(306)	
Ethnicity		
Not Hispanic	8732	88.3
Hispanic	1152	11.7
(Missing)	(92)	
Injury completeness		
Complete (AIS A)	4065	43.3
Incomplete (AIS B, C, D)	5317	56.7
(Missing)	(594)	
Category of neurologic impairment		
Tetraplegia	4946	52.4
Paraplegia	4497	47.6
(Missing)	(533)	
Hallucinogen use		
None	9911	99.3
Some	65	0.7
(Missing)	(2)	
**Continuous variable with skewed distribution**	***n* (missing)**	**Median (IQR)**
Time since injury (years)	9972 (4)	11 (5-21)
PHQ-9 total score	9103 (873)	3 (1-6)
**Continuous variable with normal distribution**	***n* (missing)**	**Mean (SD)**
Current age (years)	9972 (4)	50.5 (15.3)
SWLS total score	9732(244)	22.4 (7.5)

*Note:* AIS = American Spinal Injury Association Impairment Scale; IQR = interquartile range; PHQ-9 = Patient Health Questionnaire 9-item; SWLS = Satisfaction with Life Scale.

### Demographic, injury-related, and psychosocial factors related to hallucinogen use

**Table 2** presents the proportion of hallucinogen use by demographic, injury-related, and psychosocial factors. The following characteristics had significantly higher proportions of hallucinogen users when compared to others in the respective categories: male, non-married, unemployed, white, no rehospitalizations, those who encountered a fall, those who reported sleep disturbances, newer injuries, and younger.

**Table 2.** Proportion of hallucinogen use by demographic, injury-related, and psychosocial factors

Categorical variables	No hallucinogen use, %	Some hallucinogen use, %	*P* value
Sex			
Male	78.83	93.85	**.0008**
Female	21.17	6.15	
Education			
Less than high school	9.27	3.08	.1431
High school diploma or GED	48.59	52.31	
Greater than high school	42.15	44.62	
Income			
Less than $50k	61.24	53.33	.2152
$50k or more	38.76	46.67	
Marital status			
Married	41.77	30.77	**.0067**
Not married	34.67	53.85	
Previously married	23.57	15.38	
Employment status			
Employed/Student	27.07	47.62	**.0005**
Not employed	72.93	52.38	
Race			
White	72.01	76.69	.0021
Black	22.28	7.81	
Other, multiracial	5.72	12.50	
Ethnicity			
Not Hispanic	88.33	90.63	.5566
Hispanic	11.67	9.38	
Injury completeness			
Complete (AIS A)	43.38	35.59	.2248
Incomplete (AIS B, C, D)	56.62	64.41	
Pain interference			
None	33.58	26.16	.4319
Some	43.73	49.23	
A lot	22.70	24.62	
Rehospitalization			
None	69.69	85.48	**.0038**
Some	30.31	14.52	
Falls			
None	55.58	28.13	**<.0001**
Some	44.42	71.88	
Sleep disturbance			
Never/less than monthly	40.46	27.69	**.0323**
More than monthly	59.54	72.31	
Health status			
Good	76.16	81.54	.2961
Poor	23.84	18.46	
Continuous variables with skewed distributions	**Median (IQR)**	**Median (IQR)**	***P* value**
Time since injury, years	11.00 (5-21)	6.00 (2-12.5)	<.**0001**
Continuous variables with normal distributions	**Mean (SD)**	**Mean (SD)**	***P* value**
Current age, years	50.90 (15.27)	36.91 (10.39)	<.**0001**

*Note:* Factors with *P <* .20 are bolded and were retained for multivariable analysis. AIS = American Spinal Injury Association Impairment Scale; IQR = interquartile range.

#### Unadjusted bivariate analyses

Bivariate logistic regressions were performed to identify factors significantly related to hallucinogen use. Factors that significantly differed by hallucinogen use were sex, marital status, employment status, rehospitalization, falls, and sleep disturbance and thus were included in the subsequent multivariable logistic regression

#### Adjusted multivariable logistic regression

Results of the multivariable logistic regression indicated that age, falls, sleep disturbance, and sex were all significantly related to hallucinogen use. Results are presented in **Table 3** and indicate that for every 1-year increase in age, there was a 7% decrease in the odds of using hallucinogens (OR 0.93, 95% CI 0.91, 0.96). Individuals who reported a fall in the prior year had 3% greater odds of using hallucinogens compared to those who reported no falls (OR 3.34, 95% CI 1.82, 6.16). People with no/infrequent sleep disturbance had 50% decreased odds of using hallucinogens compared to people with regular monthly sleep disturbance (OR 0.50, 95% CI 0.278, 0.91). Finally, males had 5% greater odds of use when compared to females (OR 5.27, 95% CI 1.64, 16.95).

**Table 3.** Multivariable analysis of factors and hallucinogen use comparing the odds of using versus not using hallucinogens

Variables	Multivariable analysis			
	OR	aOR	95% CI	*P*
**Sex**				
Male	**4.094**	**5.266**	**1.636, 16.952**	.0053
Female		[Reference group]		
**Education**				
Less than high school	0.314	0.130	0.067, 1.000	.1327
High school diploma or	1.017			
GED		0.784	0.441, 1.395	
Greater than high school		[Reference group]		
**Income**				
Less than $50k	0.723	—	—	—
$50k or more		[Reference group]		—
**Marital status**				
Previously married	0.886	1.357	0.596, 3.089	.7170
Not married	**2.108**	0.978	0.502, 1.907	
Married		[Reference group]		
**Employment status**				
Employed/Student	**2.449**	1.355	0.763, 2.406	.2992
Not employed		[Reference group]		
**Injury completeness**				
Complete (AIS A)	1.386	—	—	—
Incomplete (AIS B, C, D)		—	—	—
**Pain interference**				
None	0.718	—	—	—
Some	1.038	—	—	—
A lot		[Reference group]		
**Rehospitalization**				
Some	**0.390**	0.493	0.237, 1.018	.0560
None		[Reference group]		
**Falls**				
Some	**3.198**	**3.342**	**1.817, 6.147**	<.0001
None		[Reference group]		
**Sleep disturbance**				
Never/less than monthly	**0.564**	**0.503**	**0.278, 0.912**	.0236
More than monthly		[Reference group]		
**Time since injury**	0.955	0.999	0.964, 1.035	.9529
**Current age**	0.938	**0.935**	**0.907, 0.965**	<.0001

*Note:* Bolded OR, aOR, and 95% CI are significant. OR > 1.00 indicates greater odds of hallucinogen use, OR ≪ 1.00 indicates less odds of hallucinogen use. AIS = American Spinal Injury Association Impairment Scale; aOR = adjusted odds ratio; CI = confidence interval; OR = odds ratio.

### Association between hallucinogen use and depression symptoms and QoL outcomes

Linear regressions were performed to explore the relationship between hallucinogen use and outcomes.

#### Unadjusted bivariate analyses

For SWLS, bivariate results indicated that health status, pain interference, marital status, employment, sleep disturbance, time post injury, education, sex, income, rehospitalization, and injury completeness were significant. Hallucinogen use was not significant (*P* = .2381) but was retained for the final multivariable model.

For PHQ-9, bivariate results indicated that employment, health status, pain interference, falls, sleep disturbance, injury completeness, age, sex, hallucinogen use, and rehospitalization were significant. eTable 1 presents unadjusted bivariate results.

#### Unadjusted relationship between hallucinogen use and outcomes

Without controlling for other factors, people who used hallucinogens had lower SWLS scores (*M* 21.27, SEM 0.95) compared to people who did not use them (*M* 22.39, SEM 0.08) (see eFigure 1). This difference was not statistically significant (*P* = .238), and a negligible ES of the difference was observed (ES 0.15).

Without controlling for other factors, people who used hallucinogens had higher PHQ-9 scores (*M* 7.58, SEM 0.672) compared to people who did not use (*M* 4.75, SEM 0.05) (see eFigure 2). This difference was statistically significant (*P* < .001), and a medium ES of the difference was observed (ES 0.56).

#### Adjusted multivariable linear regression analyses

**Table 4** summarizes the adjusted models for SWLS and PHQ-9, the adjusted differences in outcome scores between hallucinogen users and nonusers, and the model effects.

**Table 4.** Adjusted relationship between hallucinogen use and SWLS and PHQ-9 outcomes

			Overall adjusted model	
			SWLS	PHQ-9
***N***			9732	9103
Model ***R^2^*** adjusted			4.03e-5	0.002
Model (2, DDF)			(1, 9731)	(1, 9102)
Model ***F***			1.3992	17.671
Model ***P*** value			0.2381	<0.0001
RMSE			7.512	5.071

			Adjusted comparisons^*^	
			**SWLS**	**PHQ-9**
**No hallucinogen use - Some hallucinogen use**				
Difference			1.120	-2.832
SE difference			0.950	0.674
95% CL			-0.741, 2.982	-4.153, −1.512
Effect size^†^			0.149	0.558
		**Model effects**		
	** SWLS**		**PHQ-9**	
	***F* ratio**	***P* value**	***F* ratio**	***P* value**
Hallucinogen use	0.993	.3190	16.513	<.0001
Sex	16.960	<.0001	17.101	<.0001
Education	10.795	<.0001	—	—
Income	9.809	.0017	—	—
Marital status	72.856	<.0001	—	—
Employment status	137.875	<.0001	24.313	<.0001
Injury completeness	5.148	.0233	9.868	.0017
Pain interference	88.349	<.0001	247.338	<.0001
Rehospitalization	6.332	.0119	12.542	.0004
Falls	—	—	77.404	<.0001
Sleep disturbance	112.447	<.0001	1178.129	<.0001
Health status	576.445	<.0001	562.657	<.0001
Time post injury	111.175	<.0001	—	—
Current age	—	—	14.617	<.0001

*Note:* CL = confidence level; DDF = denominator degrees of freedom; PHQ-9 = Patient Health Questionnaire 9-item; SE = standard error; SWLS = Satisfaction with Life Scale.

* Comparisons are based on a two-sample t-test, assuming equal variance.

† Effect size computed as the mean difference divided by the root mean square error.

#### QOL

The results of the adjusted model for SWLS indicated that 24.9% of the variance in SWLS was accounted for by the combination of factors included in the model. No significant differences in SWLS scores by hallucinogen use were observed (ES 0.138, *P* = .3403).

Health status was the most significant factor related to SWLS (*F* ratio = 576.445, *P* < .0001). Other significant factors related to higher SWLS (indicating greater QOL) included those with incomplete injuries, females, those with greater than high school education, income greater than or equal to $50,000, those employed, married, self-reported good health, no pain interference, no hospitalizations, and no sleep disturbances.

#### Depressive symptoms

The results of the adjusted model for PHQ-9 indicated that 34.5% of the variance in PHQ-9 was accounted for by the combination of factors included in the model. Significant differences in PHQ-9 scores by hallucinogen use were observed (ES 0.580, *P* ≤ .0001), such that those who reported using hallucinogens had higher PHQ-9 scores, indicating greater depressive symptomatology.

The most significant factor related to PHQ-9 was sleep disturbance (*F* ratio = 1178.129, *P* < .0001). Other significant factors related to higher PHQ-9 scores (indicating greater depressive symptomatology) included those with incomplete injuries, females, those unemployed, self-reported poor health, those experiencing a lot of pain interference, one or more hospitalizations, one or more falls, and at least monthly sleep disturbances.

## Discussion

This study aimed to explore hallucinogen use among individuals with SCI and its relationship with QOL and depression. While research on hallucinogens is expanding, individuals with SCI are underrepresented in studies and clinical trials. To address this gap, we used the SCIMS NDB to gain initial insights into hallucinogen use in this population. Based on the known effects of hallucinogens on 5HT2A receptors and the alteration of 5HT receptors in individuals with SCI through neuroplasticity,^30^ we hypothesized that hallucinogen users would report fewer depressive symptoms and higher QOL than nonusers.

In our sample, 0.7% of individuals reported using hallucinogens. These results were similar to a study that looked at psychoactive substances at large in individuals with SCI and identified that less than 0.1% of its population used hallucinogens weekly and 0.4% have used hallucinogens once or twice.^22^ This proportion is notably smaller than a recent report of 3% of American adult respondents who reported using psilocybin in the past year.^31^ For our study, participants were asked about their use in the *past 3 months* whereas the aforementioned sample reported on use in the *prior year.* It is likely that as the reporting period increases, so does the percentage of people reporting use. For instance, a 2013 study that included 57,873 Americans aged 12 or older reported that 17% used hallucinogens at some point in their lifetime.^32^

After adjusting for key factors, we identified several characteristics associated with hallucinogen use, including younger age, male sex, sleep disturbances, and falls. In the general population, hallucinogen use peaks around age 19,^33^ which is notably younger than the average user age in our sample (37 years). This difference likely reflects our older study population, with an average age of 51 years. Consistent with recent data showing higher hallucinogen use among younger people,^34^ our findings showed that users were significantly younger than nonusers (37 vs. 51 years, respectively), with younger age linked to increased odds of use.

In line with prior research, our results showed that hallucinogen use was significantly associated with male sex.^35^ This may be due to males having more opportunities to try hallucinogens and a greater tendency for risky behavior compared to females.^36^,^37^ We also found that individuals who had fallen in the past year were more likely to use hallucinogens than those without falls. Although this link isn't well-documented, hallucinogens can cause dizziness, increasing the risk of falls.^38^ Additionally, hallucinogen users may engage in other risky behaviors that elevate fall risk.

Finally, sleep disturbance was strongly linked to hallucinogen use. Individuals without sleep disturbances had 50% lower odds of using hallucinogens than those with frequent disturbances. While some individuals may use hallucinogens to improve sleep, their serotonergic effects—similar to selective serotonin reuptake inhibitors—are known to prolong REM latency and reduce overall REM sleep.^39^ These findings underscore the complex relationships between hallucinogen use and various health outcomes in the SCI population, as reflected in the SCIMS NDB.

Next, we examined the association between hallucinogen use and outcomes while accounting for other potentially influential factors. The final QOL model accounted for almost 25% of the variance in SWLS scores. Factors significantly associated with higher QOL included being female, having an incomplete injury, education beyond high school, an income of $50,000 or more, being employed, being married, reporting good health, and experiencing no pain interference, rehospitalizations, or sleep disturbances. Contrary to our hypothesis, hallucinogen use was not significantly related to QOL in our population. Conflicting evidence exists regarding QOL and hallucinogen use; for example, UK data indicate that illegal drug users generally report lower SWLS scores,^40^ while a double-blind trial found that cancer patients receiving psilocybin reported increased well-being at 6 months.^4^ Our findings may have been affected by the small size of our study population, particularly those who used hallucinogens.

Our final model for depression symptoms accounted for almost 35% of the variance in depression symptoms. We found a significant difference in depression symptoms between hallucinogen users and nonusers, such that users reported greater depressive symptoms. Other factors significantly related to greater depression symptoms included incomplete injury, sex (female), unemployment, self-reported poor health, high levels of pain interference, rehospitalizations, falls, and sleep disturbances. While we initially predicted that hallucinogen use would be linked to fewer depressive symptoms, our model did not support this. It's possible that individuals with depression use hallucinogens to self-medicate. Additionally, the diverse mechanisms of action within the hallucinogen class can lead to varying effects on depression and suicidality. For example, LSD has been associated with an increased risk of depression and suicidal thoughts, whereas ecstasy/MDMA tends to reduce the likelihood of depression and suicidal ideation.^41^ Though evidence supports the overall minimal and short lasting adverse effects of hallucinogen use, negative symptoms such as intense anxiety, dysphoria, disconnection, and visual disturbances have been reported that may persist in a small number of cases, typically with higher dosages.^42^–^45^ Experience of prolonged hallucinogenic adverse effects may contribute to depressive symptoms. Due to SCIMS reporting limitations, we could not identify which specific hallucinogens were used or their dosages, which may help explain why hallucinogen users in our study had higher depressive symptoms.

## Limitations

We recognize several limitations in this study. First, it is an unpowered, exploratory study focused on potential relationships between hallucinogen use and various factors. Future fully powered studies can build on our preliminary findings. Additionally, the cross-sectional nature of the dataset prevents us from establishing causal relationships between hallucinogen use and outcomes. Given this, we are unable to control for baseline depression scores for individuals with SCI both prior to their SCI and prior to the use of hallucinogens. Longitudinal studies would provide stronger evidence of these causal links. The response criteria for WHO ASSIST 2 also limits our research, as we are unable to understand dosing patterns and how this could possibly affect outcomes. Additionally, the broad hallucinogen definition, or what individuals might perceive to fall in this category, limits our understanding of what substances are specifically related to which outcomes.

We did not assess pain severity or interference in this analysis. As these variables are associated with mood and QOL in this population, future work is needed to determine the potential relationship between these factors and hallucinogen use in individuals with SCI.^46^ Furthermore, it is important to note that there is also a bidirectional relationship between many of the variables in relation to depression. This includes sleep disturbances and mood disorders, such that sleep disturbances increase the likelihood of mood disorders, and mood disorders increase the likelihood of sleep disturbances.^47^ The same bidirectional relationship occurs within pain and depression and is theorized to occur as part of the effect of glutamate, an excitatory neurotransmitter in the process of both chronic pain and depression.^48^

Our research was also limited by the small number of hallucinogen users in the SCIMS NDB, likely due to underreporting. Many hallucinogens are classified as Schedule I drugs by the FDA, which may discourage individuals from disclosing their use during SCIMS interviews. However, as states continue to enact psychedelic reform legislation—36 states introduced related bills in 2022, mostly concerning psilocybin—reporting may improve.^49^ As the research landscape expands and stigma decreases, we may see improved reporting, enhancing our understanding of the benefits and risks associated with hallucinogen use in this population.

## Conclusion

This study provides a foundational analysis of hallucinogen use in relation to depression and QOL among individuals with SCI. Future research should focus on specific hallucinogens, such as LSD, mescaline, and psilocybin, to better understand their individual effects on various psychosocial outcomes. Additionally, fully powered longitudinal studies are needed to explore causal relationships between hallucinogen use and psychosocial outcomes in SCI, including motivations for use and user experiences. Building on this work will yield deeper insights into the effects of hallucinogens on this specific group and enhance our overall understanding of their impact.

## Supplementary Material




