# Supplementary material for: Association of Hallucinogen Use With Psychosocial Outcomes: Secondary Analysis of the Spinal Cord Injury Model Systems Database
Source: Top Spinal Cord Inj Rehabil. 2025 Nov 18;31(4):160–74. doi: 10.46292/sci24-00082 (PMC12629206; doi:10.46292/sci24-00082)
Supplement: Supplementary file 1 [file i1945-5763-31-4-160_s01.pdf]

eTable 1. Unadjusted bivariate analysis for each factor and outcome (SWLS, PHQ-9)

	SWLS			PHQ-9		
	M	**SEM**	**Model *P* value**	***M***	**SEM**	**Model *P* value**
**Hallucinogen use**						
None	22.390	0.076	.2381	4.747	0.053	<.0001
Some	21.270	0.946		7.579	0.672	
**Sex**						
Male	22.319	0.086	.1016	4.588	0.060	<.0001
Female	22.624	0.166		5.421	0.116	
**Education**						
Less than high school	21.567	0.255	<.0001	5.533	0.178	<.0001
High school diploma or GED	21.923	0.111		4.953	0.077	
Greater than high school	23.133	0.118		4.351	0.082	
**Income**						
Less than $50K	21.405	0.105	<.0001	5.221	0.075	<.0001
$50K or more	24.271	0.132		4.156	0.093	
**Marital status**						
Married	23.980	0.116	<.0001	4.396	0.082	<.0001
Not married	20.937	0.127		4.967	0.090	
Previously married	21.687	0.155		5.125	0.110	
**Employment status**						
Employed/Student	25.052	0.145	<.0001	3.720	0.102	<.0001
Not employed	21.496	0.089		5.100	0.063	
**Injury completeness**						
Complete (AIS A)	22.270	0.119	.1305	4.457	0.830	<.0001
Incomplete (AIS B, C, D)	22.501	0.104		4.965	0.073	
**Pain interference**						
None	24.569	0.126	<.0001	2.789	0.084	<.0001
Some	22.616	0.110		4.507	0.074	
A lot	18.755	0.154		8.312	0.104	

(continues)

This supplementary digital material accompanies
Top Spinal Cord Inj Rehabil 2025;31(4);160-174
© 2025 American Spinal Injury Association
www.asia-spinalinjury.org
doi: 10.46292/sci24-00082

eTable 1. Unadjusted bivariate analysis for each factor and outcome (SWLS, PHQ-9) (*cont.*)

	**SWLS**			**PHQ-9**		
	M	**SEM**	**Model *P* value**	***M***	**SEM**	**Model *P* value**
**Rehospitalization**						
None	22.911	0.091	<.0001	4.415	0.063	<.0001
Some	21.152	0.139		5.551	0.097	
**Falls**						
None	22.665	0.102	<.0001	4.062	0.071	<.0001
Some	22.031	0.114		5.645	0.079	
**Sleep disturbance**						
Never/Less than monthly	24.264	0.118	<.0001	2.143	0.074	<.0001
More than monthly	21.113	0.967		6.63	0.063	
**Health status**						
Good	23.863	0.082	<.0001	3.709	0.056	<.0001
Poor	17.632	0.147		8.323	0.103	
	**SWLS**			**PHQ-9**		
**Continuous variables**	**Estimate**	**SEM**	**Model *P* value**	**Estimate**	**SEM**	**Model *P* value**
Time since injury (years)				-0.027	0.004	<.0001
Current age (years)	0.029	0.005	<.0001	-0.013	0.003	<.0001

Note: *P* value threshold <.20 for retaining. AIS = American Spinal Injury Association Impairment Scale; PHQ-9 = Patient Health Questionnaire–9; SEM = standard error of the mean; SWLS = Satisfaction With Life Scale.
