# Supplementary material for: Association of Hallucinogen Use With Psychosocial Outcomes: Secondary Analysis of the Spinal Cord Injury Model Systems Database
Source: Top Spinal Cord Inj Rehabil. 2025 Nov 18;31(4):160–74. doi: 10.46292/sci24-00082 (PMC12629206; doi:10.46292/sci24-00082)
Supplement: Supplementary file 2 [file i1945-5763-31-4-160_s02.pdf]

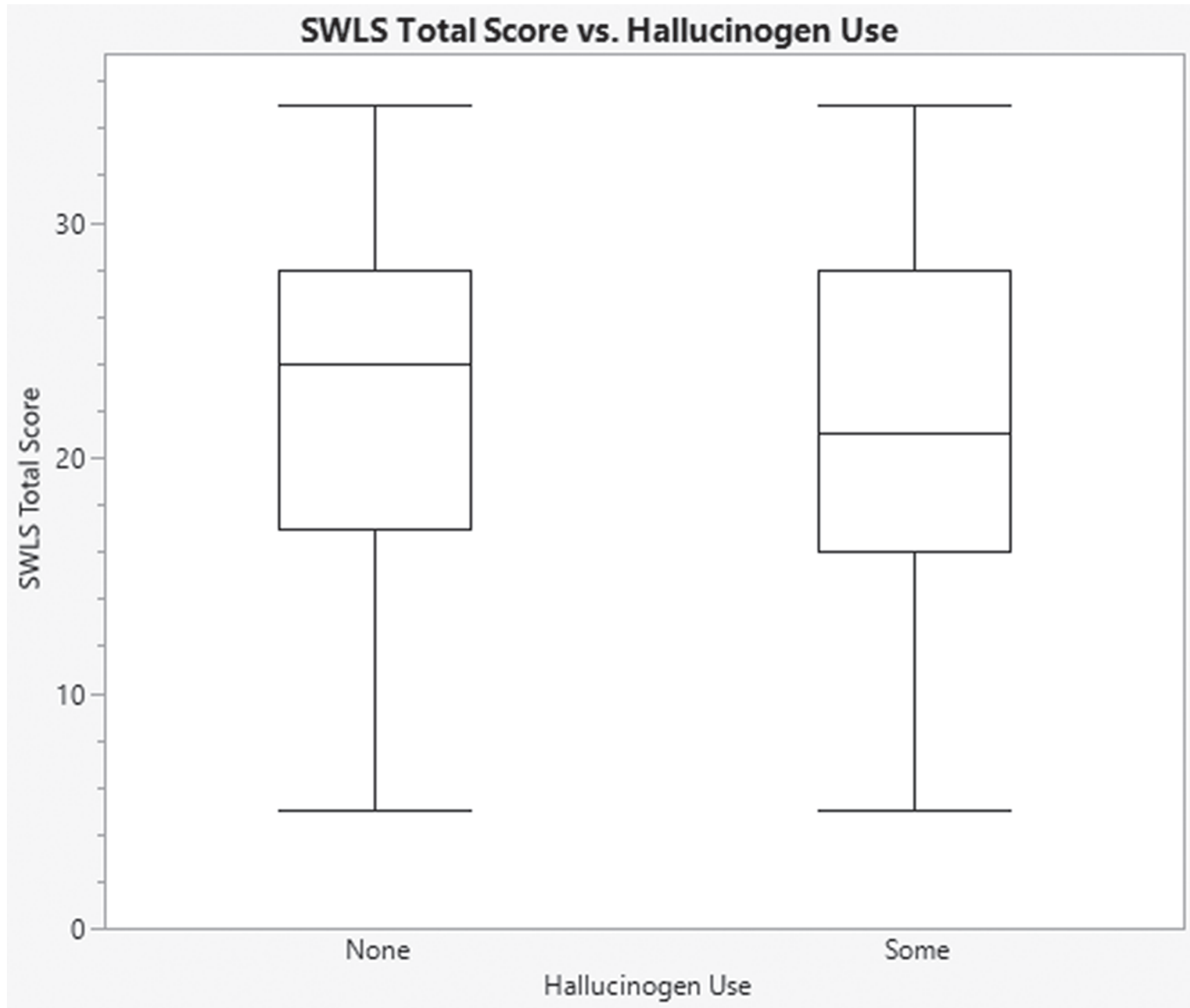

eFigure 1. Satisfaction With Life Scale (SWLS) scores stratified by hallucinogen use.

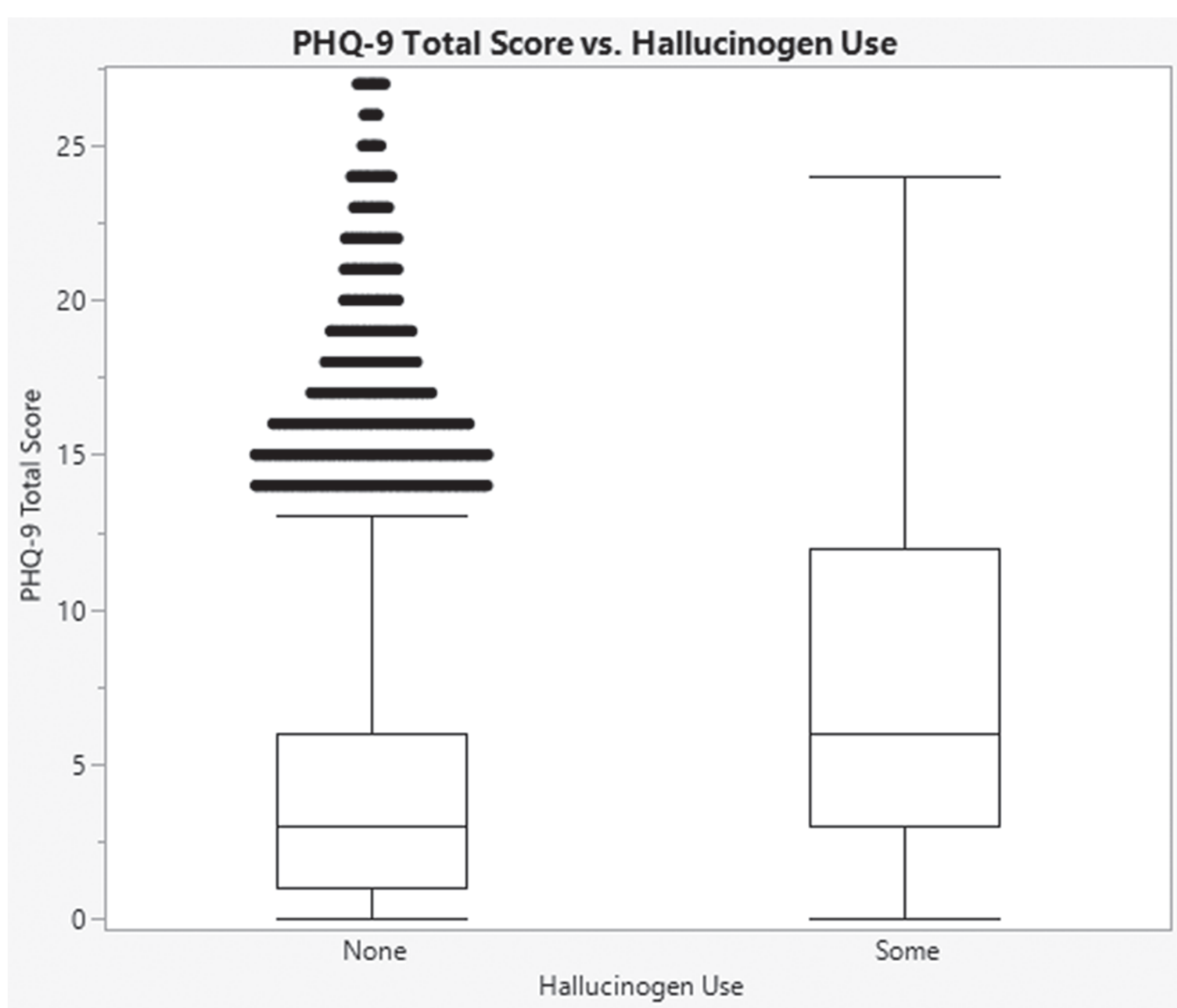

eFigure 2. Patient Health Questionnaire–9 (PHQ-9) scores stratified by hallucinogen use.

This supplementary digital material accompanies
Top Spinal Cord Inj Rehabil 2025;31(4):160-174
© 2025 American Spinal Injury Association
www.asia-spinalinjury.org
doi: 10.46292/sci24-00082
